# Streamlined Assessments are Integral to Operationalizing Resilience

**DOI:** 10.1007/s00267-025-02200-7

**Published:** 2025-07-16

**Authors:** Garrett Watson, Michael Deegan, Luke Hogewood, Andrew Jin, Holden Keebaugh, Frank Randon, Igor Linkov

**Affiliations:** 1 U.S. Army Corps of Engineers, Engineer Research and Development Center (ERDC), Vicksburg, MS USA; 2 U.S. Army Corps of Engineers, Institute for Water Resources (IWR), Alexandria, VA USA; 3 U.S. Army Corps of Engineers, HQ, Washington, DC USA

## Abstract

As infrastructure systems face growing challenges from threats and increasing complexity, resilience has become a critical focus in sustaining essential services and managing risks. However, existing frameworks for assessing resilience are often impractical for owner-operators of critical infrastructure like dams and watersheds due to their complexity, extensive data requirements, and high costs. The failure of the Oroville Dam spillway in 2017, which resulted in mass evacuations and extensive damages, underscores the urgency for practical assessment strategies. This perspectives piece highlights the development of a streamlined resilience assessment approach co-developed with the U.S. Army Corps of Engineers’ Savannah District. Unlike traditional assessments that often become “one-and-done” exercises, our framework leverages simplified metrics, expert elicitation, and existing data to create adaptable and reproducible measures. By using a modified Analytical Hierarchy Process (AHP) to align resilience metrics with stakeholder priorities, this approach balances the need for comprehensive data with operational feasibility. Key insights suggest that streamlined assessments are integral to bridging the gap between resilience theory and practice, making it easier for decision-makers to evaluate and improve baselines consistently. Future efforts should emphasize metrics that are interpretable, prioritizable, and replicable to foster a culture of continuous improvement in resilience planning.

## Introduction

Resilience, broadly defined as the ability of systems to withstand, adapt to, and recover from disruptions, has become a central concept in ensuring the sustainability and functionality of critical infrastructure (Linkov et al. 2014). In watershed management, resilience is a particularly important consideration due to the varied and unpredictable nature of challenges, which can range from long-term droughts to sudden, large-scale infrastructure failures. One notable example is the Oroville Dam spillway failure, which in 2017 forced the evacuation of 180,000 people and caused nearly $1.1 billion in damages. As infrastructure becomes more complex and threats change unpredictably, the need for resilient systems is growing (Normand 2019).

Watershed resilience poses unique challenges due to the inherent complexity of these socio-environmental systems. Watersheds are not just about managing water—they are vast interconnected systems where natural processes intersect with human infrastructure. Owner-operators of dams and other critical infrastructure within watersheds need to balance multiple system goals, including flood management, power generation, water availability, transportation, navigability, and recreation (McManamay et al. 2016). For instance, in the United States, managing a watershed involves coordinating between the U.S. Army Corps of Engineers (USACE), the Environmental Protection Agency, State Water Resource Agencies, and numerous stakeholders with water-rights claims. The involvement of these multiple agencies adds layers of regulatory complexity and operational challenges, as each stakeholder brings different objectives to the table.

Evolving environmental conditions introduce new and unpredictable variables into the equation. Variations in temperature, shifting precipitation patterns, and an increase in extreme weather events place additional strain on existing water systems (Wilby and Keenan 2012). These pressures require dam operators to manage not only the immediate demands on water resources but also to prepare for long-term shifts in watershed behavior. Extended dry periods may necessitate improved drought management, while more intense storms heighten the risk of flooding, adding stress to existing flood management systems (Watts et al. 2011). As infrastructure networks become more intricate; owner-operators face a growing need to manage these evolving demands efficiently while maintaining service reliability. The coordination between various functions—flood control, hydropower, irrigation, and recreational uses—requires a level of adaptability that is becoming increasingly difficult to manage without robust, comprehensive frameworks.

The complexity of watershed management is not just about the physical infrastructure but also the multiplicity of stakeholders involved. Each entity—whether federal, state, or local—has different mandates and goals, as well as differences in institutional priorities and values. For example, while the United States Army Corp of Engineers (USACE) might focus on maintaining navigability and flood control, local communities might prioritize recreational access or environmental protection (Wood et al. 2012). These competing objectives often complicate decision-making processes and make it difficult to implement comprehensive resilience strategies. Moreover, stakeholders can include private industries like power/water utilities that have vested interests in how the water is managed. These different perspectives must be balanced when implementing resilience measures, making the coordination of goals critical but challenging.

Traditionally, watershed resilience has focused on the analysis of ecological or hydrological components of the system (Lane et al. 2023; Rathburn et al. 2018). However, as the push to improve resilience of critical infrastructure systems grows, the overall social-economic systems of watersheds requires understanding of resilience beyond just the impact on the natural environment. In response to these needs, researchers have emerging trends to understand the social-ecological-hydrological systems, water resources engineering and management, nature-based solutions, and resilience thinking are incorporated into planning, watershed resilience has grown (Miralles-Wilhelm et al. 2023).

As such, efforts to operationalize watershed resilience have begun to emerge, especially through qualitative assessments. A wide range of work has been done to merge natural and social science in the realm of socio-hydrology and hydrosocial analysis (Wesselink et al. 2017). (Nemec et al. 2013) assessed the performance of the Platt River social-ecological system’s resilience along nine factors, including ecological modularity, innovation, and social diversity, each measured through stakeholder feedback (Nemec et al. 2013). Koebele et al. described a pilot program in Colorado to “align watershed restoration and risk mitigation with community and economic development goals” through a multi-jurisdictional approach to improve disaster recovery (Koebele et al. 2020). Several factors have pushed owner-operators of dam infrastructure to develop metrics beyond qualitative assessments. First, federal and international initiatives, such as the White House Grand Pathways Resilience initiative or NATO layered resilience directives have pushed requirements to measure resilience and implement investments to improve it (Roberts et al. 2023; NATO HQ SACT 2023). Deliberate attacks on dam infrastructure, such as the destruction of the Kakovkha dam, have highlighted the critical nature of resilience (Glanz et al. 2023). To meet this impetus, this work develops a resilience assessment that is streamlined for reproducibility and repeatability.

We found, through co-developing a new resilience methodology with a large system operator, that there is a strong need for streamlined, reproducible measures of resilience. We explored resilience metrics through the requirements of an owner-operator of dams in the United States—the U.S. Army Corps of Engineers’ Savannah District. The Savannah District operates three major dams, the Strom Thurmond Dam, Richard B. Russell Dam, and Hartwell Dam, which have a combined nameplate electricity generation of over 1.4 Gigawatts of electricity, and control flooding for major urban areas such as Savannah and Augusta, Georgia. These dams also serve other community and ecosystem functions, such as serving as a drinking water source for numerous communities, sustaining diverse ecosystems, and allowing over 1 million recreational visitors each year.

This forum paper will first introduce challenges described by existing resilience frameworks, including some key feedback received by stakeholders. We then describe using the resilience matrix as a tool for more structured feedback and expert elicitation. Next, we discuss a simplified methodology to incorporate feedback given by experts and convert this feedback into metrics that can be used by decision makers. Finally, three key attributes of streamlined assessments—replicability, prioritizable, and interpretability—that serve as key features to guide streamlined resilience assessments.

## Current Resilience Assessments are too Complex for Consistent Use

Recent developments in resilience assessments aim to provide a comprehensive overview of concept across different dimensions, but they are often impractical for owner-operators. Off-the-shelf assessments do exist, such as the Integrated Resilience Planning Framework (IRPF), Federal Infrastructure Resilience Assessment (FIRA), Regional Resilience Assessment Program (RRAP), and IN-CORE. However, these can be cost, time, and logistically difficult for owner-operators to perform. The IRPF, for instance, provides a framework for assessing resilience across social, economic, physical, and environmental domains. However, it requires extensive data collection and collaboration across multiple sectors (Cybersecurity & Infrastructure Security Agency 2024). While it delivers a holistic picture of resilience, its implementation proves cumbersome and resource intensive. One critical gap in this framework is the need for continuous data updates. Without regular, ongoing input, these assessments quickly become outdated, especially as threats and infrastructural needs evolve.

Through our work with Savannah District, we convened two workshop meetings with emergency managers, operational managers, civil engineers, hydraulic engineers, and natural resources officers at both the local district and federal management levels. In our conversations with district personnel, these assessments, especially FIRA and RRAP frameworks, were previously considered to provide in-depth assessments but required an overwhelming volume of metrics to track (Cybersecurity & Infrastructure Security Agency, 2021). Thus, these assessments were viewed as typically conducted once and not regularly updated, leading to a “one-and-done” perspective on these assessment types. A critical inability to continually track resilience improvements is a significant limitation, making it difficult for owner-operators to evaluate progress or adjust strategies over time (Cybersecurity & Infrastructure Security Agency, 2021). Similarly, IN-CORE, another comprehensive framework, focuses heavily on modeling and simulation to predict how infrastructure will respond to various disruptions (Lee et al. 2019). While it offers detailed insights into potential vulnerabilities, this was also found to require substantial computational resources and a high level of technical expertise, making it difficult to apply consistently across different contexts or for operators with limited resources. In the face of these challenges, amongst others, decision-makers have been shown to be unlikely to choose more complex decision making tools, and rather default to relying on heuristics (Siders and Pierce 2021).

Instead, district personnel and federal program managers expressed the need for reproducible and streamlined metrics. Both the district personnel and federal program managers expressed concerns that an extensive resilience analysis would be a “one-and-done”. While these would be able to create a preliminary baseline, they would not be able to show the benefits of newly implemented projects or initiatives that were meant to improve resilience from the initial assessment. Because these in-depth assessments would not be able to be performed on short timelines, they would not be able to inform decision-making as new initiatives and projects changed the resilience landscape.

Experts at the workshops highlighted the need for threat-agnostic frameworks to measure resilience, citing a key distinction between risk management and resilience analytics. The threat-vulnerability-consequence model of many of the existing assessments require performing threat models, such as flood models, to understand resilience. However, this causes both an operational and a theoretical problem. First, performing flood modeling and scenarios can be time and computationally expensive, requiring modelers to analyze numerous potential scenarios. Second, these can leave blind spots where scenarios might not be able to capture the types of failures a watershed could experience.

## Bridging the Resilience Data Gap Requires a Shared Vision for Approaching Resilience

Resilience has been defined differently throughout the times and by different people. Resilience managers within the district had key ideas of what types of programs fell under resilience. However, past research has shown that many of these ideas can conflate resilience with traditional risk-management. Thus, we asked stakeholders to map key resilience assets to the resilience matrix framework. The resilience matrix is a tool that has been used to measure capabilities and metrics for all phases of an adverse event (absorb, recover, and adapt) along the key domains on which decision have to be made (physical, social, and information) (Linkov et al. 2013). We found that many of the system capabilities that our workshop participants were focused on were within the absorb phase of an adverse event, with fewer measurable capabilities identified in the recover and adapt phases (Table 1).

**Table 1:** Capabilities identified by resilience personnel at the watershed and federal level mapped to the phases of an adverse event (absorb, recover, adapt) and the domains of a complex system (physical, information, and social)

	Absorb	Recover	Adapt
Physical	System Performance/Functionality System Reliability Robustness Consequences of failure System Vulnerability Hazard Mitigation Measures Redundancy Back-up Systems Emergency Resources	Recovery Time Temporary Facilities Recovery Resources	Adaptive Capacity Infrastructure Condition
Information	Failure Detection Systems Hazard Forecasting Risk Assessment/Data Emergency Planning Mitigation Planning Disaster Propagation Models	Recovery Tracking Data Models for Recovery Scenarios Recovery Planning	Post-disaster data collection Adaptation Planning Plan Improvements
Social	Emergency Staffing Emergency Support Agreements Community Communication Staff Emergency Training	Community Recovery Assistance Contractor Agreements Recovery Agreements	Training Exercises Community Education Improved Legislation

Each of the metrics defined in the NIST inventory are then mapped to these key capabilities

Resilience data metrics can be incredibly complex and difficult to navigate. The NIST inventory of resilience metrics identifies over 56 frameworks, with more than 1000 individual metrics (Walpole et al. 2021). Because these frameworks require specific metrics to be calculated, they often duplicate data collection requirements for very similar metrics needed for other reporting. Using this mapping exercise, dam operators, emergency planners, and other key personnel identified existing data that was required for non-resilience programs to be integrated into resilience planning. For example, to measure system performance during an adverse event, data collected for reporting to the National Inventory of Dams could be easily compiled to show how dams would perform under different flooding scenarios. This approach minimized the burden on stakeholders by answering as many resilience metrics as possible with readily available information.

## Identifying Stakeholder Priorities and Streamlining Results

During our workshops and conversations, a consistent concern was measuring baselines against threats that would be impossible to recover from quickly. As an emergency manager stated, preventing a “12-foot flood” would never be feasible to meet the 1–3-year goals of the organization. Certain system priorities were more important to not just operational managers but the local community as well. These priorities were local to the requirements of the Savannah District, and not necessarily generalizable to other districts. The stakeholders mentioned that “we need to include navigation in the assessment, as this is a primary critical function, following hydropower, water and FRM [Flood Risk Management]”. Other priorities were also identified, such “floodplain communities are a big concern of ours when it comes to FRM.”

Thus, resilience metrics must be flexible to the priorities of each organizational unit that is responsible for maintaining operations in that domain. Different watersheds will have different priorities, and stakeholder knowledge is a key resource to identifying what may or may not be important. In our workshop, we implemented an elicitation process where stakeholders at the dam were asked to score specific metrics on a Likert-like scale of 1 (Low Priority) to 5 (High Priority), as shown in Table 2. This simplified scoring mechanism allowed us to quickly gather subjective and objective input from experts without requiring them to participate in a more time-intensive data collection process. By combining this expert elicitation with existing data, we were able to create a comprehensive resilience assessment without overwhelming stakeholders with additional workload. This process was done across multiple critical functions (Flood Risk Management and Hydropower) and proved easy to replicate the process for each.

**Table 2:** Resilience Scorecard Template—Values were scored from 1 through 5 across the main phases of resilience (Absorb, Recover, Adapt) and throughout the key components of complex systems (Physical, Information, and Social)

Flood risk management			
Resilience matrix	Absorb	Recover	Adapt
Physical	4.5	4.3	1.5
Information	4.3	1.7	3.5
Social	4.0	2.0	4.3

Measurements of resilience are only as useful as their ability to inform resilient decision making. To provide a simplified score, we used a modified Analytical Hierarchy Process (AHP), a common multi-criteria decision analytic tool that quantifies the final performance of a system by multiplying expert-elicited weights with measurable metrics (Vaidya and Kumar 2006). While decision makers may not get as much clarity on the specific performance of individual components as more-robust metrics, this tool allows for identifying how different system priorities can impact the final evaluation of critical infrastructure, systems, and/or missions.

This data is presented to stakeholders in the same form as the resilience matrix (Table 2). As such, each cell in the matrix becomes a criterion for resilience of a system. In this way, operational managers expressed that such values could be interpretable and actionable when discussing how different projects may impact resilience overall. Thus, this acts as a “scorecard” for the current state of resilience in a project and can highlight the benefits and tradeoffs of implementing a new project.

## Future Work Needs to Engage Stakeholders to see if Metrics are Reasonable to Implement

**Fig. 1:**
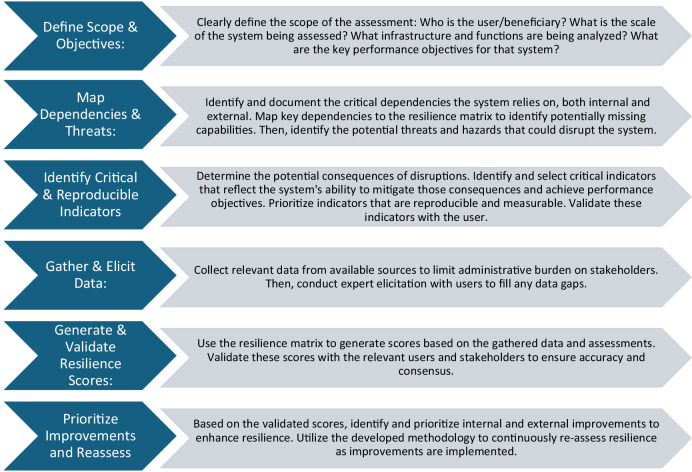
Generalized framework of streamlined resilience assessments

The methodology we present in this paper to provide a simplified resilience metric is in no way meant to be comprehensive or indicative of the exact best-practice for assessing resilience. Rather, it provides just one framework that can be used to assess resilience over time (Fig. 1). However, it follows a general systematic approach to ensure the reproducibility of resilience assessments over time.

These steps provided a streamlined version of traditionally defined resilience assessments which could be performed over time by assessment teams with reduced workloads on project managers themselves. Rather, this preliminary exploration of methods with the Savannah District highlighted three principles for actionable resilience in watersheds:

1. **Replicability**—assessments need to be streamlined so that they can be consistently measured throughout the lifespan of various projects.
2. **Prioritizable**—metrics need to meet the priorities of the watersheds they are trying to measure
3. **Interpretability**—resilience metrics need to be able to be interpreted by the managers and other key personnel of a watershed. Metrics need to clearly show how resilience changed within a system because of a project being implemented.

Reducing the burden of resilience assessments will greatly improve the buy-in to resilience. As one federal planner put it, “we don’t want an academic exercise in resilience”, but operationalizable metrics that can inform decision making. This framework offers a preliminary, practical alternative to more comprehensive resilience assessments but is not intended to replace them. While it simplifies decision-making and improves usability, it does so at the cost of some technical depth. Moreover, objectively evaluating whether one resilience metric is “better” than another remains a challenge. This approach is meant to give stakeholders a clearer view of potential gaps in resilience planning, rather than provide definitive answers.

Future work needs to develop resilience metrics that balance the comprehensive data requirements of existing metrics with the operational feasibility of consistently assessing resilience as projects get implemented or community priorities change.

## Data Availability

No datasets were generated or analysed during the current study.
